# Predicting community walking after stroke

**DOI:** 10.3389/fstro.2025.1523242

**Published:** 2025-04-09

**Authors:** Richard A. W. Felius, Michiel Punt, Natasja C. Wouda, Marieke Geerars, Sjoerd M. Bruijn, Jaap H. van Dieën

**Affiliations:** ^1^Research Group Lifestyle and Health, Utrecht University of Applied Sciences, Utrecht, Netherlands; ^2^Department of Human Movement Science, Amsterdam Movement Sciences, Vrije Universiteit Amsterdam, Amsterdam, Netherlands; ^3^Center of Excellence for Rehabilitation Medicine, UMC Utrecht Brain Center, University Medical Center Utrecht, Utrecht University and De Hoogstraat Rehabilitation, Utrecht, Netherlands; ^4^Department of Neurorehabilitation, De Hoogstraat Rehabilitation, Utrecht, Netherlands; ^5^Physiotherapy Department Neurology, Axioncontinu, Rehabilitation Center de Parkgraaf, Utrecht, Netherlands

**Keywords:** accelerometers, stroke recovery, stroke rehabilitation, gait quality, prediction, walking ability, community walking, walking performance

## Abstract

**Introduction:**

A key element of personalized stroke rehabilitation is early prediction of an individual's potential to walk in the community.

**Objective:**

We aim to determine the predictive value of patient characteristics, clinical test results, and Inertial Measurement Units (IMU) based balance, clinical gait and daily-life measures, measured at admission and discharge in clinical stroke rehabilitation, for community walking 6 months after stroke.

**Methods:**

Data were collected from people after stroke during clinical rehabilitation and at 6 months post stroke. The assessment during rehabilitation consisted of an IMU-based 2-min walk test (2MWT), three IMU-based balance tests, an IMU-based measurement of gait in daily life, and several standard clinical tests, including the Berg Balance Scale, Barthel Index, Functional Ambulation Categories, Motricity Index (MI), and Trunk Control Test (TCT). At 6-months, gait in daily life was measured with an IMU for two consecutive days. From this measurement, three gait features were calculated, namely the strides per day, and average and maximum gait speed. We assessed the predictive value of IMU-based balance, gait, and daily-life measures, the clinical tests and patient characteristics at admission and discharge for predicting daily-life measures at 6 months after stroke with univariate ordinary least squares regression. Subsequently, significant predictors were included in a multivariate ordinary least squares regression.

**Results:**

Thirty-five individuals after stroke were included. Ordinary least squares regression analysis indicated that age, gait features and strides per day at admission and discharge had significant predictive value for the step count at 6 months. For the average and maximum gait speed in daily life at 6 months, the 2MWT gait speed, TCT, MI and the baseline average and maximum gait speed in daily life were significant predictors. Multivariate analysis indicated that the outcomes at admission had more predictive value than the outcomes at discharge, with adjusted *R*^2^ values for the strides per day, average and maximum gait speed models of 0.60, 0.42, and 0.53, respectively.

**Conclusions:**

Age, trunk stability (TCT), affected leg strength (MI), and the clinical and daily-life gait had predictive value for community walking 6-months after stroke. Future research with a larger sample size is required to refine these findings.

## 1 Introduction

Walking problems are common in people after stroke, leading to decreased mobility, independence, and overall quality of life (Lord et al., 2004; Park and Kim, 2019; Grau-Pellicer et al., 2019). These problems are due to a variety of impairments, such as muscle weakness, spasticity, and reduced coordination (Li et al., 2018). The nature and intensity of the impairments are directly linked to the type and severity of the stroke, as well as the affected brain area (Payabvash et al., 2018). Recovery after stroke typically follows a logarithmic pattern, with the most significant improvements in walking ability occurring in the first few weeks after stroke (Kwakkel et al., 2004). These improvements can be attributed to spontaneous recovery and clinical rehabilitation, which can both induce neural plasticity as well as adaptive behaviors.

After 6 months, recovery reaches a critical point from which individuals can likely only improve functional gait by adopting compensation strategies, i.e., functional adaptations to overcome impairments (Stanhope et al., 2014; Cirstea and Levin, 2000; Buurke et al., 2008).

The differences in impairments, recovery rates and compensation strategies make the stroke population a heterogenous group with a wide variety of movement patterns (Felius R. A. W. et al., 2024; Balaban and Tok, 2014). A consequence of the variety within the stroke population is that there is no single intervention that caters to the needs and circumstances of each individual. Therefore, personalized rehabilitation, in which interventions are tailored to the unique impairments, needs and goals, is of paramount importance to achieve the best possible outcomes in stroke rehabilitation (Chang, 2022; Moore et al., 2022).

One important aspect of personalized rehabilitation is accurate prediction of an individual's potential to walk in the community after rehabilitation. Such predictions allow clinicians to focus intervention strategies and manage patient expectations more effectively (Moons et al., 2009). There are various ways to define “walking in the community”, with the simplest approach being a questionnaire to assess participation in community activities (Lord et al., 2004; Ada et al., 2009). However, the subjective nature of questionnaires can lead to biased outcomes. These are difficult to predict, as they might misrepresent an individual's true walking capacity, defined as the distance an individual is capable of walking during a given period of time (Robinson et al., 2011). A more objective method is to characterize the level of walking in the community via the number of strides per day, in which a higher step count reflects a higher level of community walking (Fulk et al., 2017). To get a better understanding of walking in the community after stroke, other measures should be explored as well. Two potentially interesting measures are the average and maximum gait speed in the community. These might provide a different perspective of someone's ability to walk in the community, as they may be less affected by environmental factors than step count. Moreover, evidence suggests that clinical gait speed is directly linked to community walking, therefore it is imaginable that gait speed in the community is an indicator of community walking as well (Fulk et al., 2017; An et al., 2015; Bijleveld-Uitman et al., 2013; Vive et al., 2021; Grau-Pellicer et al., 2019).

Previous work indicated several predictors that influence walking in people after stroke (Fulk et al., 2017; Wouda et al., 2024; Kollen et al., 2005). Fulk et al. (2017) used a cross-sectional analysis to predict community walking, defined in terms of step count. They found that gait speed, movement impairments, and balance were significant predictors for walking in the community (Fulk et al., 2017). The review of Wouda et al. (2024) focused on early predictors of walking independence after stroke. They concluded that trunk stability while sitting and the strength of the affected leg, both assessed on the 9th day after stroke had predictive value for independent walking after stroke (Wouda et al., 2024). Lastly, research by Kollen et al. (2005) investigated the factors associated with changes in functional gait within the 1st year after stroke. They found that improvements in strength, balance and synergism were linked to improvements in functional gait.

Beside the previously indicated predictors, evidence suggests that gait features, i.e., gait characteristics, such as asymmetry and variability, are associated with walking abilities as well (Wonsetler and Bowden, 2017; Punt et al., 2016; Patterson et al., 2008). Therefore, it is conceivable that these also have predictive value for the capacity of people after stroke to walk in the community. However, to the best of our knowledge there is no study that evaluated the predictive value of these features for community walking after stroke.

The objective of this study was to determine the predictive value of clinical tests, balance features, clinical gait features, daily-life measures and patient characteristics, measured during clinical rehabilitation, on the ability to walk in the community 6-months post stroke. Community walking was measured with three different gait features, namely the number of strides per day, the average gait speed and the maximum gait speed in daily life. Understanding which variables have predictive value for community walking is crucial, as they can support accurate goal setting, manage patient expectations and may help clinicians to tailor interventions to expected level of recovery.

## 2 Materials and methods

### 2.1 Participants

Individuals after stroke in the acute and sub-acute phase were recruited in two clinical rehabilitation centers in the Netherlands between January 1, 2021, and July 1, 2023. The inclusion criteria were as follows: (1) participants aged 18 years or older; (2) capable of understanding and signing the informed consent document; (3) able to understand and perform simple tasks; (4) first-ever or recurrent stroke; and (5) able to walk at least 8 m within 2 min without physical assistance (FAC 3). Participants provided written informed consent before participating. Participants were excluded if either the initial or follow-up measurement was missing. During rehabilitation, participants received care depending on the individual needs, including physiotherapy, speech therapy, and occupational therapy. The Medical Ethical Review Committee of Utrecht approved the protocol (20-462/C).

### 2.2 Instruments

**Figure 1:**
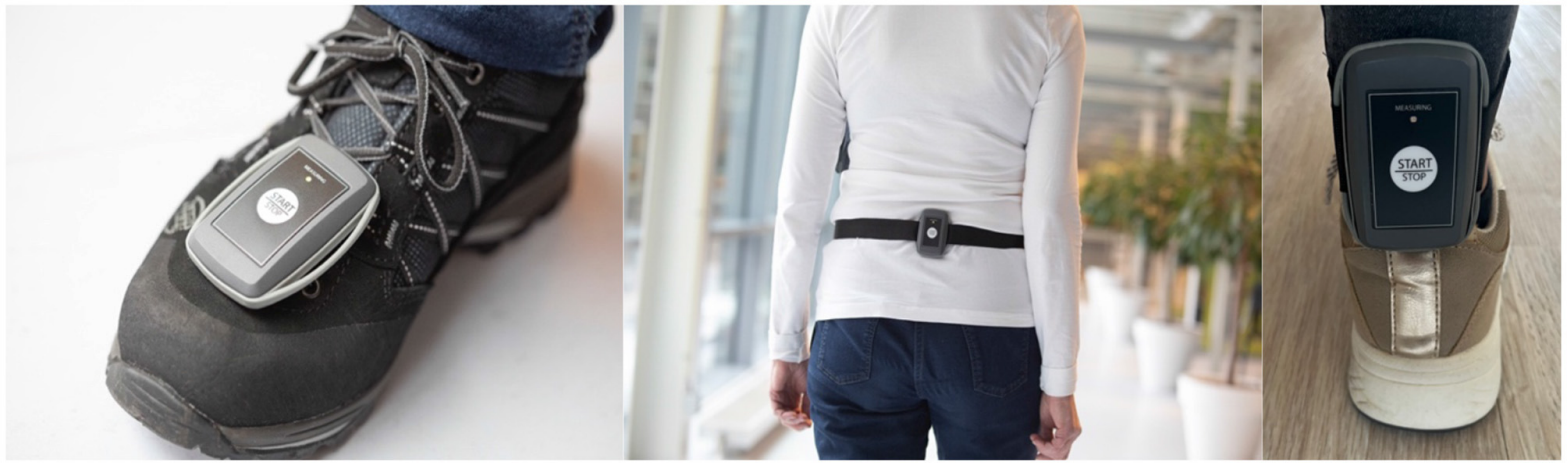
Image of the Inertial Measurements Units (IMUs). The IMUs were used to measure clinical gait during a 2MWT, postural sway during various balance conditions and gait in daily life.

Inertial Measurements Units (IMUs, Aemics b.v. Oldenzaal, The Netherlands) were used in this study. IMUs can be used to measure a variety of human activities, including balance and gait (Gujarathi and Bhole, 2019; Renggli et al., 2020; Kim and Eng, 2004; Ohta et al., 2023; Jarvis et al., 2022; Felius et al., 2023, 2022a; Felius R. et al., 2024; Alsubaie et al., 2019; Ghislieri et al., 2019; Patel et al., 2020; Jung et al., 2020). The IMUs consisted of a triaxial accelerometer and gyroscope and could measure up to 104 samples per second with a maximum of 16g and 2,000 rad/s (Figure 1).

### 2.3 Protocol

The assessment was administered every 3 weeks during clinical rehabilitation, from admission to discharge. At 6 months after stroke, one follow-up assessment was administered in the participant's home environment. The assessment during rehabilitation and the follow-up assessment consisted of four and two parts, respectively, which are described below. Prior to the initial assessment, demographic and stroke-specific characteristics were collected, including sex, age, stroke type and side, and pre-morbid walking aid use. All assessments were administered by an experienced physiotherapist or trained research assistant.

#### 2.3.1 Assessment during clinical rehabilitation

*Part 1.1*: Participants walked for 2 min at a self-selected speed on a fourteen-meter walking path (Jarvis et al., 2022). Three IMUs were used to collect data from the left and right foot, as well as the lower back. The IMUs were attached using elastic bands. The IMUs measured with a sampling rate of 104 samples/s. Participants were allowed to walk with a walking aid during the walking test.

*Part 1.2*: Participants completed three balance conditions to measure postural sway. The balance conditions were (1) Sitting unsupported on a balance cushion with feet touching the ground and knees in a 90 degrees angle for 60 s; (2) Standing with feet in a self-selected position for 60 s; (3) Standing with eyes closed and feet in a self-selected position for 30 s (Felius et al., 2022a). The sensor was placed at the back, at the level of T7 during sitting and L5 during standing, of the upper body and whole body, respectively.

*Part 1.3*: Several standard clinical tests were administered, including the Berg Balance Scale (BBS) (Berg et al., 1992) and Barthel Index (BI) (Collin et al., 1988), the Functional Ambulation Categories (FAC), both with and without a walking aid (Holden et al., 1984), and the Motricity Index (MI) (Demeurisse et al., 1980).

*Part 1.4:* The participant's' ability to ambulate in daily life was monitored over two consecutive days using one IMU. Participants were instructed to always wear the sensor, except while sleeping or showering. The IMU was fixed to the ankle of the paretic leg. If no paretic leg could be identified, the IMU was attached to the right ankle. The IMU measured with a sampling rate of 52 samples/s. The location and sampling rate were adjusted in comparison to the 2MWT to minimize the risk of sensor loss and enhance battery life. Moreover, a single sensor was used instead of three, enhancing the feasibility of the measurements and reducing the risk of sensor loss. The measurement automatically stopped after ~2 days when the IMU's battery depleted. No specific instructions or goals were given to the participants regarding the measurement. Participants walked with and without walking aid, depending on their needs.

#### 2.3.2 Follow-up assessment

*Part 2.1:* Participants were contacted by telephone to schedule a semi-structured interview and to receive instructions for the IMU-based sensor measurements (part 2). If participants could not independently complete the interview or the IMU measurement, they were advised to seek help from a family member or caregiver.

*Part 2.2:* An IMU sensor was sent to the participants via post. Instructions provided guided them to secure the IMU around the ankle of the paretic leg. If no paretic leg could be identified, individuals were instructed to attach the IMU to the right ankle. The IMU was worn during normal daily activities for two consecutive days to measure ambulation in daily life. To initiate the measurements, participants were directed to press the start button on the sensor (Figure 1). A special code was implemented within the sensor's system to prevent accidental deactivation. Participants were instructed to always wear the sensor, except while sleeping or showering. The measurement period concluded automatically after ~2 days when the IMU's battery depleted. Subsequently, participants were instructed to return the sensors via post.

### 2.4 Data processing

The collected IMU data underwent digital processing on a custom-made online platform, where data were processed and stored. The IMU data were processed by first resampling the data to 100 samples/s for the balance and gait measurement, and to 50 samples/s for the daily-life gait measurement. Next the gyroscope offset was corrected by subtracting the previously collected offset during a static measurement. Last, clinical gait, balance and daily-life measures were calculated.

#### 2.4.1 Clinical gait (part 1.1)

Gait was described using seven gait features. The following four gait features were calculated using conventional calculation methods: speed [m/s], asymmetry, smoothness, and variability [s] (Ghislieri et al., 2019; Patel et al., 2020; Jung et al., 2020; Berg et al., 1992). These four gait features were selected because they are reliable and represent different aspects of gait (Tasseel-Ponche et al., 2022; Garcia et al., 2021). The other three gait features were calculated with a Variational AutoEncoder (VAE). A VAE is a generative model that uses deep learning to learn a low dimensional representation of the data, while minimizing data loss (Felius R. et al., 2024; Kingma and Welling, 2019). These gait features were included, as previous research indicated good clinimetric properties and that they contain different information than the conventional gait features (Felius R. et al., 2024). A detailed description of the VAE and the seven gait features is provided in the Supplementary Appendices S2, S4.

#### 2.4.2 Balance (part 1.2)

The postural sway data of the three balance conditions was described using one feature per measurement, namely: path length. The path length was computed as the length of the postural sway trajectory determined from the IMU [m/s^2^] (Felius et al., 2022b; Alsubaie et al., 2019; Ghislieri et al., 2019). A higher path score indicates more movement during the balance assessment, which is associated with a worse gait. A detailed description of path length is provided in the Supplementary Appendix S1.

#### 2.4.3 Daily-life gait (part 1.4 and 2.2)

The data of the IMU-based measurement in daily life were split up into parts of 10 s before applying a previously trained convolutional neural network with long-short term memory to identify episodes of gait in daily life (Felius et al., 2023). The model was trained on a balanced dataset containing walking at gait speeds between 0.5 and 5 km/h, among other activities, such as sitting, lying, standing, and standing kitchen work. The model achieved an accuracy of 0.93 indicating an excellent ability to identify gait. The following three features were calculated: number of strides per day, and average and maximum gait speed. The maximum gait speed was determined from the 95th percentile of the speed distribution during the measurement. A detailed description of the calculation of the daily-life measures is provided in the Supplementary Appendix S3.

### 2.5 Statistical analysis

Ordinary Least Squares (OLS) regression was employed to predict the daily-life measures (dependent variables) at 6-months after stroke (Mauskopf, 2005). The collected clinical gait, balance and daily-life measures, clinical tests and patient characteristics both at admission and discharge were used as predictors. The predictors were transformed into *z-*scores to detect outliers. Absolute *z-*scores higher than 3 were removed from the analysis. For each daily-life gait feature, a separate univariate OLS was created per predictor. Statistical significance was determined by a *p-*value of < 0.05. If a significant relationship between the predictor and the response variable was established, the following assumptions were evaluated to ensure the validity of the results: (1) linearity; (2) normality of error terms; and (3) homoscedasticity. A multivariate OLS analysis was conducted using significant predictors identified at admission and discharge. A forward selection method was employed, beginning with the most significant predictor. Additional predictors were subsequently added one at a time, and each was retained in the multivariate model it resulted in an increase of the adjusted *R*^2^. This process ensured that each new predictor enhanced the explanatory power of the model. Both the univariate and multivariate OLS were conducted with the available data. The final multivariate OLS was evaluated and described using the adjusted *R*^2^, the model's *p-*value and the *F*-statistic.

## 3 Results

### 3.1 Characteristics

Longitudinal data of 35 people after stroke were collected (Table 1). On average, participants were discharged after 62 days (SD 64, range 8-379). Participants improved on all the standard clinical tests (BBS, BI, FAC, MI, TCT) from admission to discharge. Of the IMU-based balance, clinical gait and daily-life gait measurements, only the strides per day showed a notable increase during rehabilitation. The outcomes of the dependent variables at follow-up were comparable to the outcomes at discharge.

**Table 1:** Predictors at admission and discharge and dependent variables at follow-up.

		**Predictors**				**Dependent variables**	
		**Rehabilitation**				**Follow-up**	
		**Admission**	***N***	**Discharge**	***N***	**6-Months**	***N***
**Demographics**							
Sex	Male/Female	18/17	35				
Age in years	μ (σ)	69.9 (10.1)	35				
Pre-morbid walking aid	Yes/no	4/31	35				
Stroke type	Ischemic/hemorrhagic/other	26/6/3	35				
Stroke side	Left/right/other	17/10/8	35				
Time after stroke (weeks)	μ (σ)	4.2 (2.5)	34	13.3 (9.6)	35		
**Clinical tests**							
BBS	μ (σ)	38.0 (18.1)	32	47.9 (11.8)	29		
BI	μ (σ)	13.6 (5.6)	35	18.8 (1.8)	31		
FAC	μ (σ)	2.1 (1.9)	34	3.6 (1.6)	30		
MI paretic leg	μ (σ)	82.8 (21.4)	33	91.1 (14.3)	29		
TCT		91.1 (17.2)	33	98.3 (5.5)	30		
**Balance conditions**							
USIT [m/s^2^]	μ (σ)	25.7 (9.4)	31	21.4 (8.8)	28		
EO [m/s^2^]	μ (σ)	28.9 (10.8)	27	23.2 (6.8)	26		
EC [m/s^2^]	μ (σ)	20.4 (7.3)	25	22.8 (15.4)	27		
**Clinical gait**							
Walking aid [%]	Yes/No	41/59	22	35/65	27		
Speed [m/s]	μ (σ)	0.81 (0.24)	22	0.82 (0.27)	27		
Asymmetry	μ (σ)	0.96 (0.16)	21	1.05 (0.41)	27		
Variability	μ (σ)	0.06 (0.02)	21	0.06 (0.02)	26		
Smoothness	μ (σ)	0.44 (0.11)	22	0.4 (0.16)	27		
VAE 1	μ (σ)	0.08 (0.68)	21	0.11 (0.75)	24		
VAE 2	μ (σ)	0.19 (0.77)	21	0.12 (0.96)	24		
VAE 3	μ (σ)	−0.81 (0.52)	21	−0.78 (0.53)	24		
**Gait in daily life**							
Wearing time [h]	μ (σ)	27.1 (8.9)	22	26.4 (7.3)	23		
Strides per day^a^	μ (σ)	2,785 (1,786)	22	4,083 (1,984)	23	3,874 (2,536)	35
Average gait speed [m/s]	μ (σ)	0.45 (0.13)	22	0.46 (0.1)	23	0.43 (0.13)	35
Maximal gait speed [m/s]^b^	μ (σ)	0.87 (0.17)	21	0.88 (0.15)	23	0.79 (0.2)	35

^a^Strides per day was calculated as the number of strides divided by the wearing time per day.

^b^The maximum gait speed was calculated as the 95% gait speed.

### 3.2 Prediction models

A univariate OLS was created for each predictor for the strides per day (Table 2), average gait speed (Table 3) and maximum gait speed (Table 4), both for admission and discharge. None of the included predictors was significant for all three response variables. Subsequently, all predictors with significant predictive value were added to the multivariate models (Table 5).

**Table 2:** Univariate prediction of strides per day in daily life 6-months after stroke.

	**Strides per day in daily life**							
		**Admission**				**Discharge**		
	β **(SE)**	**[–CI, CI]**	***T***	***P***	β **(SE)**	**[–CI, CI]**	***T***	***P***
**Demographics**								
Sex	−408 (867)	[−2,174, 1,357]	−0.5	0.64				
Age [years]^*^	–**98 (40)**	**[**–**180**, –**16]**	–**2.4**	**0.02**				
Pre-morbid walking aid	−2,506 (1,296)	[−5,143, 131]	−1.9	0.06				
Time after stroke weeks	−49.6 (178.5)	[−413, 314]	−0.3	0.78				
**Clinical tests**								
BBS	15 (25)	[−36, 67]	0.6	0.55	64 (35)	[−8, 137]	1.8	0.08
BI	85 (77)	[−71, 242]	1.1	0.28	326 (233)	[−150, 803]	1.4	0.17
FAC	260 (227)	[−202, 722]	1.1	0.26	439 (269)	[−111, 991]	1.6	0.11
MI paretic leg	22.24 (21.3)	[−21.1, 65.6]	1	0.3	24.5 (32)	[−41.9, 91.0]	0.8	0.46
TCT	19.4 (26.6)	[−34.9, 73.6]	0.7	0.47	46.2 (77)	[−113, 205]	0.6	0.56
**Balance conditions**								
USIT [m/s^2^]	−30.06 (50.3)	[−133, 72.8]	−0.6	0.55	−44.2(50)	[−146.1, 57.7]	−0.9	0.38
EO [m/s^2^]	−9.02 (45.9)	[−103.5, 85.5]	−0.2	0.85	−14.9(65)	[−150, 120.5]	−0.2	0.82
EC [m/s^2^]	−103 (67)	[−241.9, 34.5]	−1.6	0.13	−3.9 (28)	[−62.9, 55.2]	−0.1	0.89
**Clinical gait**								
Speed [m/s]	2,869 (2,320)	[−1,970, 7,710]	1.2	0.23	1,015 (1,476)	[−2,025, 4,055]	0.7	0.5
Asymmetry	1,736 (3,730)	[−6,071, 9,543]	0.5	0.65	−557 (1,067)	[−2,755, 1,640]	−0.5	0.61
Smoothness	2,542 (5,161)	[−8,223, 13,308]	0.5	0.63	−761 (2,711)	[−6,344, 4,822]	−0.3	0.78
Variability	−21,949.27 (23,880.4)	[−71,931, 28,033]	−0.9	0.37	−17,192 (20,296)	[−59,083, 24,697]	−0.8	0.41
VAE 1	779 (886)	[−1,075, 2,633]	0.9	0.39	**1,817 (499)**	**[780, 2,853]**	**3.6**	**< 0.01**
VAE 2^*^	–**2,132 (633)**	**[**–**3,458**, –**805]**	–**3.4**	**< 0.01**	–**1,218 (470)**	**[**–**2,194**, –**243]**	–**2.6**	**0.02**
VAE 3	−645 (1,175)	[−3,106, 1,815]	−0.5	0.59	−977 (815)	[−2,669, 714]	−1.2	0.24
**Gait in daily life**								
Average gait speed [m/s]	−1,084 (4,122)	[−9,684, 7,515]	−0.3	0.8	1,384 (4,430)	[−7,829, 10,597]	0.3	0.76
Maximum gait speed [m/s]	−1,303.15 (3,358.9)	[−8,333.4, 5,727.1]	−0.4	0.7	1,299 (2,744)	[−4,445, 7,043]	0.5	0.64
Strides per day^*^	**0.85 (0.24)**	**[0.35, 1.35]**	**3.5**	**< 0.01**	**0.42 (0.2)**	**[0.01, 0.83]**	**2.1**	**0.04**

A significant relationship (p < 0.05) was marked in bold.

Predictors that had a significant relationship with the response variable at admission and discharge were marked with a ^*^.

**Table 3:** Univariate prediction of the average gait speed in daily life 6-months after stroke.

	**Average gait speed in daily life**							
		**Admission**				**Discharge**		
	β **(SE)**	**–CI, CI**	***T***	***P***	β **(SE)**	**–CI, CI**	***T***	***P***
**Demographics**								
Sex	−0.65 (4.6)	[−9.9, 8.6]	−0.1	0.89				
Age [years]	0.12 (0.2)	[−0.3, 0.6]	0.5	0.6				
Pre-morbid walking aid	6.98 (7.1)	[−7.4, 21.3]	1	0.33				
Time after stroke weeks	−1.49 (0.9)	[−3.3, 0.4]	−1.6	0.11				
**Clinical tests**								
BBS	−0.04 (0.1)	[−0.3, 0.2]	−0.3	0.79	0.29 (0.2)	[−0.2, 0.7]	1.3	0.2
BI	0.32 (0.4)	[−0.5, 1.15]	0.8	0.43	0.74 (1.3)	[−2.0, 3.5]	0.5	0.59
FAC	−1.29 (1.2)	[−3.7, 1.14]	−1.1	0.29	1.76 (1.1)	[−0.5, 4.1]	1.6	0.13
MI paretic leg	**0.22 (0.1)**	**[0.0, 0.4]**	**2.1**	**0.04**	0.16 (0.2)	[−0.2, 0.5]	0.9	0.38
TCT	0.18 (0.1)	[−0.1, 0.5]	1.4	0.17	**1.06 (0.4)**	**[0.3, 1.9]**	**2.7**	**0.01**
**Balance conditions**								
USIT [m/s^2^]	0.08 (0.2)	[−0.4, 0.6]	0.3	0.75	−0.27 (0.3)	[−0.9, 0.4]	−0.8	0.4
EO [m/s^2^]	0.37 (0.2)	[−0.0, 0.7]	2	0.05	0.26 (0.4)	[−0.5, 1.1]	0.7	0.51
EC [m/s^2^]	0.35 (0.3)	[−0.2, 0.9]	1.3	0.21	−0.07 (0.2)	[−0.4, 0.3]	−0.4	0.71
**Clinical gait**								
Speed [m/s]	–**19.25 (8.22)**	**[**–**36.4**, –**2.1]**	–**2.3**	**0.03**	−9.8 (8.6)	[−27.5, 7.9]	−1.1	0.27
Asymmetry	−0.23 (14.1)	[−29.8, 29.4]	0	0.99	1.52 (6.3)	[−11.5, 14.6]	0.2	0.81
Smoothness	−18.14 (19.6)	[−59.0, 22.8]	−0.9	0.37	−4.03 (16.1)	[−37.1, 29.0]	−0.3	0.8
Variability	111 (92)	[−81, 305]	1.2	0.24	141 (107)	[−79, 361]	1.3	0.2
VAE 1	4.33 (3.3)	[−2.6, 11.3]	1.3	0.21	−4.6 (3.7)	[−12.3, 3.1]	−1.2	0.23
VAE 2	−4.56 (2.9)	[−10.6, 1.5]	−1.6	0.13	−0.75 (3.2)	[−7.5, 6.0]	−0.2	0.82
VAE 3	5.45 (4.4)	[−3.7, 14.6]	1.2	0.23	5.88 (4.9)	[−4.3, 16.1]	1.2	0.25
**Gait in daily life**								
Average gait speed [m/s]^*^	**35.42 (11.1)**	**[12.1, 58.7]**	**3.2**	**< 0.01**	**66.51 (23.4)**	**[17.8, 115.2]**	**2.8**	**0.01**
Maximum gait speed [m/s]^*^	**24.53 (8.1)**	**[7.6, 41.5]**	**3**	**0.01**	**35.5 (16.0)**	**[1.9, 69.0]**	**2.2**	**0.04**
Strides per day	−0.0 (0.0)	[−0.0, 0.0]	−1	0.33	−0.0 (0.0)	[−0.0, 0.0]	−0.5	0.6

Significant variables (p < 0.05) were marked in bold.

Predictors that had a significant relationship with the response variable at admission and discharge were marked with a ^*^.

**Table 4:** Univariate prediction of the maximum gait speed in daily life 6-months after stroke.

	**Maximum gait speed in daily life**							
		**Admission**				**Discharge**		
	β **(SE)**	**–CI, CI**	***T***	***P***	β **(SE)**	**–CI, CI**	***T***	***P***
**Demographics**								
Sex	−5.19 (6.7)	[−18.8, 8.4]	−0.8	0.44				
Age [years]	0.13 (0.3)	[−0.6, 0.8]	0.4	0.71				
Pre-morbid walking aid	8.98 (10.5)	[−12.4, 30.4]	0.9	0.4				
Time after stroke weeks	−2.4 (1.3)	[−5.1, 0.3]	−1.8	0.08				
**Clinical tests**								
BBS	0.05 (0.2)	[−0.3, 0.4]	0.2	0.81	0.56 (0.3)	[−0.1, 1.2]	1.8	0.09
BI	0.63 (0.6)	[−0.6, 1.9]	1.1	0.3	1.26 (1.8)	[−2.5, 5.0]	0.7	0.5
FAC	−1.01 (1.8)	[−4.6, 2.6]	−0.6	0.58	2.06 (2.5)	[−3.0, 7.1]	0.8	0.41
MI Paretic leg	**0.4 (0.1)**	**[0.1, 0.7]**	**2.8**	**0.01**	0.48 (0.3)	[−0.0, 1.0]	1.9	0.07
TCT	0.31 (0.2)	[−0.1, 0.7]	1.6	0.11	**1.54 (0.5)**	**[0.4, 2.7]**	**2.8**	**0.01**
**Balance conditions**								
USIT [m/s^2^]	0.29 (0.3)	[−0.4, 1.0]	0.8	0.41	−0.51 (0.5)	[−1.5, 0.5]	−1.1	0.29
EO [m/s^2^]	0.41 (0.3)	[−0.1, 0.9]	1.6	0.12	0.56 (0.5)	[−0.5, 1.6]	1.1	0.28
EC [m/s^2^]	0.51 (0.4)	[−0.3, 1.3]	1.3	0.19	−0.14 (0.2)	[−0.6, 0.4]	−0.6	0.59
**Clinical gait**								
Speed [m/s]	−23.5 (12.7)	[−50.0, 3.1]	−1.8	0.08	−9.45 (11.2)	[−32.5, 13.6]	−0.8	0.41
Asymmetry	10.36 (20.9)	[−33.4, 54.1]	0.5	0.63	6.21 (8.1)	[−10.4, 22.8]	0.8	0.45
Smoothness	−20.2 (29.4)	[−81.5, 41.1]	−0.7	0.5	−29.15 (19.8)	[−70.0, 11.7]	−1.5	0.15
Variability	157 (137.9)	[−131, 446]	1.1	0.27	141 (134)	[−134, 417]	1.1	0.3
VAE 1	3.7 (5.1)	[−7.0, 14.4]	0.7	0.48	−6.12 (4.9)	[−16.3, 4.1]	−1.2	0.23
VAE 2	–**8.93 (4.1)**	**[**–**17.5**, –**0.3]**	–**2.2**	**0.04**	−1.78 (4.3)	[−10.7, 7.1]	−0.4	0.68
VAE 3	11.59 (6.2)	[−1.5, 24.7]	1.9	0.08	6.35 (6.6)	[−7.4, 20.1]	1	0.35
**Gait in daily life**								
Average gait speed [m/s]	44.65 (22.5)	[−2.3, 91.6]	2	0.06	65.14 (34.3)	[−6, 136]	1.9	0.07
Maximum gait speed [m/s]	**55.95 (15.8)**	**[22.9, 89.0]**	**3.5**	**< 0.01**	44.0 (23.3)	[−4.7, 92.7]	1.9	0.07
Strides per day	−0.0 (0.0)	[−0.0, 0.0]	−1.1	0.27	−0.0 (0.0)	[−0.0, 0.0]	0	1

Significant variables (p < 0.05) were marked in bold.

**Table 5:** Multivariate prediction of daily-life measures 6-months after stroke.

		**Admission**				**Discharge**		
	β **(SE)**	**–CI, CI**	***T***	***P***	β **(SE)**	**–CI, CI**	***T***	***P***
**Strides per day**								
Age [years]	−59.8 (38.1)	[−141, 22]	−1.6	0.14	−41.1 (28.8)	[−102, 20]	−1.4	0.19
VAE 1	−1,704	[−2,928, −480]	−3.0	< 0.01	1,037 (547)	[−136, 2,211]	1.9	0.08
VAE 2					201 (420)	[−700, 1,101]	0.48	0.64
Strides per day	0.74 (0.25)	[0.212, 1.270]	3.0	0.01	0.47 (0.22)	[−0.01, 0.94]	2.1	0.05
**Average gait speed**								
TCT					0.65 (0.44)	[−0.26, 1.57]	1.5	0.15
Speed [m/s]	−7.7 (8.6)	[−25.9, 10.5]	−0.9	0.38				
Average gait speed [m/s]	62.6 (20.6)	[19, 106]	3.0	< 0.01	60.9 (25.6)	[7, 114.7]	1.8	0.03
**Maximum gait speed**								
MI paretic leg	0.38 (0.25)	[−0.16, 0.92]	1.5	0.16				
TCT					1.58 (0.34)	[0.88, 2.29]	4.6	0.01
VAE 2	−7.8 (16.0)	[−16.0, 0.5]	−2.0	0.06				
Maximum gait speed [m/s]	52.6 (16.0)	[18.2, 87.1]	3.3	0.05				

#### 3.2.1 Univariate prediction of strides per day

Three variables at admission and three variables at discharge were predictive for the strides per day in daily life 6-months post stroke. Age, VAE 2 and strides per day at admission, and VAE 1, VAE 2 and strides per day at discharge had predictive value for the strides per day at 6 months post stroke. None of the clinical-tests and balance-conditions predictors were significant. Evaluation of the OLS assumptions indicated no violation (Supplementary Figures S5.1, S5.2).

#### 3.2.2 Univariate prediction of average gait speed

Four variables at admission and three variables at discharge were significantly related to the average gait speed in daily life 6 months post stroke. The MI paretic leg, clinical gait speed, average gait speed, and maximum gait speed had significant predictive value for the average gait speed 6 months post stroke. Evaluation of the OLS assumptions indicated no violation (Supplementary Figures S5.3, S5.4).

#### 3.2.3 Univariate prediction of maximum gait speed

Three variables at admission and one variable at discharge had significant predictive value for the maximum gait speed in daily life. The MI at admission and TCT at discharge were positively related to the maximum gait speed. The VAE 2 at admission was negatively related to the maximum gait speed. Evaluation of the OLS assumptions indicated no violation (Supplementary Figures S5.5, S5.6).

#### 3.2.4 Multivariate prediction of daily-life measures

The coefficients of the predictors that improved the multivariate model are described in Table 5. The final Adjusted *R*^2^ values for the models for strides per day, average and maximum gait speed models with variables measured at admission were 0.60 [*F*_(3, 18)_ = 9.6, *p* < 0.01], 0.41 [*F*_(3, 19)_ = 5.1, *p* = 0.01] and 0.53 [*F*_(3, 14)_ = 7.3, *p* < 0.01], respectively. The adjusted *R*^2^ values for predictors measured at discharge for the strides per day, average and maximum gait speed were 0.53 [*F*_(4, 14)_ = 6.2, *p* < 0.01], 0.36 [*F*_(3, 18)_ = 6.7, *p* < 0.01], and 0.40 [*F*_(1, 29)_ = 21.3, *p* < 0.01], respectively.

## 4 Discussion

We determined the predictive value of patient characteristics, standard clinical tests and IMU-based balance, clinical gait and daily-life measures at admission and discharge for the ability to walk in the community 6 months post stroke. None of the included variables had significant predictive validity for all three measures (strides per day, average gait speed, and maximum gait speed) of walking in the community. Per dependent variable, only a few of the included variables had predictive value. Of these, the results were mostly inconsistent between admission and discharge. To further improve prediction accuracy other factors, such as cognitive, behavioral and environmental factors, should be considered as well.

For the number of strides at 6 months, we found age, number of strides at admission and discharge, and gait features at admission and discharge to be predictive. The effect of age is in line with previous research which indicated that age has a negative relationship with recovery after stroke (Bagg et al., 2002; Kugler et al., 2003). The predictive value of the stride count at admission and discharge may reflect that individuals with a higher walking capacity at onset also have a higher walking capacity at the follow-up measurements, but may also reflect a trait to be physically active which was preserved over the study duration. Lastly, gait features indicated by features obtained with a Variational AutoEncoder were significant. Presumably, gait impairments result in a lower locomotion efficiency (Brodie et al., 2017; Kramer et al., 2016; Blokland et al., 2021, 2023), i.e., higher energy expenditure during walking, causing individuals to walk less. None of the conventional clinical-test outcomes had significant predictive validity on the step counts in the community. These results are in contrast with the conclusions of Fulk et al. (2017) who were able to discriminate between home and community ambulators using conventional clinical tests, such as the 6MWT and the BBS. The differences might be explained by the large differences in sample size, since they had a sample size which was ~12 times larger. Moreover, they used walking in the community as a categorical variable. This however has several limitations. First, a large amount of information is lost by transforming a continuous outcome measure into a dichotomous outcome measure, disregarding all variance within the defined groups. Second, individuals that are close to but on opposite sides of the cut-off points appear different, however might be very similar. Therefore, in this study we decided to use continuous outcomes of community walking.

The predictors for the average and maximum walking speed in daily life were comparable, as for both trunk stability (TCT), affected leg strength (MI paretic leg), clinical gait speed (2MWT), and gait-speed in daily life at admission and discharge had significant predictive value. The predictors were mostly not consistent between admission and discharge. This makes it unclear if the results are caused by differences in test-scores between admission and discharge not reflected in daily-life walking or are due to a measurement error. Notably, multivariate analysis indicated that variables measured at admission explained more variance in the daily-life measures at 6 months than variables measured at discharge. Nonetheless, a considerable amount of variation remains unexplained for both admission and discharge. A possible explanation for this finding might be that the severity of impairments at the time of admission is a critical determinant of long-term outcomes despite rehabilitation efforts Another explanation could be that the variation between individuals is lower at discharge, as balance and gait are often criteria for discharge. This reduced variation might have complicated the estimation of coefficients. Clinical gait features were poor predictors for the daily-life measures. Therefore, a simple evaluation of gait with a 2MWT, might result in inaccurate estimations of someone's ability to ambulate in the community (Waters and Mulroy, 1999; Takayanagi et al., 2019).

We found one gait feature, VAE2, obtained with a Variational AutoEncoder to have high predictive value for the number of strides per day both at admission and discharge. This finding indicates that gait impairments during clinical rehabilitation are somehow related to the walking at a later stage, which is not captured in the conventional gait features, such as gait speed and asymmetry. In previous research, VAE2 was also found to have good psychometric properties (test-retest ICC: 0.85; difference stroke-healthy: *P* < 0.01). VAE2 was computed with a type of deep learning, which makes it difficult to identify which aspect of gait this variable reflects. In a previous study, we created an online tool, accessible via http://edu.nl/p3kv4, to visualize what effect a change in the VAE2 (L2) outcome has on raw sensor data. Visual inspection suggests that the variable is a combination of stride length and stride smoothness, with higher and smoother peaks in the anterior-posterior and vertical direction. Future research might further explore alternative data-driven methods to process IMU-data, or other movement data, as these might be more suitable to extract the predictive information from complex multidimensional data than conventional gait features.

None of the included features were predictive of all three measures of daily-life measures. This finding was unexpected, as all three daily-life measures were anticipated to be related. For example, we hypothesized that individuals capable of walking faster would also walk relatively more compared to those with slower walking speeds. However, we were unable to confirm this assumption, suggesting that these measures are different within the stroke population. Further research is needed to better understand the relationship the associations among different daily-life gait measures.

The strength of this study is that a variety of different types of data was collected that provided new insight into the relationship between walking capacity and walking in daily life after stroke. This study also has several limitations. The first limitation is the small sample size (*N* = 35) of the follow-up data. Moreover, not all individuals were able to conduct all tests at admission and discharge resulting in some missing values. Given these facts, in combination with the number of evaluated predictors and the risk of false positives, the results should be interpreted with caution. Nevertheless, some predictors were significant both at admission and discharge which makes it more likely that the results are accurate. Secondly, we measured daily-life gait for only two consecutive days, which might not be sufficient to accurately capture daily-life gait for individuals after stroke given the day-to-day variance. Moreover, we did not collect information about the use of walking aids in daily life, which could have influenced outcomes as well. Thirdly, in this study, only data of individuals after stroke in clinical rehabilitation were collected, therefore the conclusions are not applicable to the whole stroke population. Fourthly, the IMUs were placed on the forefoot during the 2MWT, whereas they were positioned on the ankle during daily-life measurement. Consequently, gait speed recorded during the 2MWT may differ from that observed in daily life. Küderle et al. (2022) confirmed that sensor placement affects single-stride errors and variability. However, they also reported that the average error across multiple strides remains relatively stable (Küderle et al., 2022). Since our study relies on aggregated gait speed, we assume that sensor location had minimal impact on comparability. Nevertheless, the inconsistent placement of IMUs between the clinical and daily-life settings should be acknowledged as a limitation of the study. Last, ordinary least squares regression is a technique that assumes a linear relationship between the predictors and response variables. This assumption may not always hold true and should be evaluated in future studies with larger sample sizes.

## 5 Conclusion

We aimed to assess the extent to which patient characteristics, observed during rehabilitation, could predict community walking 6 months post stroke. We discovered that age, clinical gait features, and the amount of walking in daily life had predictive value for the number of steps 6 months after stroke. Furthermore, we found affected leg strength (MI paretic leg), trunk stability (TCT) and speed during a 2-min walk test (2MWT) and everyday walking speed to be predictive for daily-life gait speed. Future research with a larger sample size is required to confirm these findings.

## Data Availability

The data analyzed in this study is subject to the following licenses/restrictions: Non-sensitive data will be made available after publication. Requests to access these datasets should be directed to richard.felius@hu.nl.
