# Supplementary material for: Predicting community walking after stroke
Source: Front Stroke. 2025 Apr 9;4:1523242. doi: 10.3389/fstro.2025.1523242 (PMC12802673; doi:10.3389/fstro.2025.1523242)
Supplement: Supplementary file 1 [file Data_Sheet_1.docx]

**Supplements**

**Supplements S1: Calculation of IMU-based balance features**

All calculations were done using Python (version 3.7.3.). First, the signal was down sampled to 100 samples per second. Second, the gyroscope was corrected by subtracting the gyroscope offset. Third, to account for the movement during transitional periods, the first and last 2 seconds of each trial were excluded from further analysis. Fourth, the accelerometer and gyroscope data were aligned with the vertical (VT)(upward: positive), medial-lateral (ML) (right: positive), and anterior-posterior (AP) axes (anterior: positive) and corrected for the effects of gravity. Lastly, the accelerometer data was filtered with a 0.4Hz third-order high-pass Butterworth filter to minimise noise as an effect of respiration. The path length was calculated as the total length of the trajectory (equation 1).

$Path length \left[ m/s^{2} \right]=\Sigma\sqrt{\left( a_{AP} \right)^{2}+\left( a_{ML} \right)^{2}}$ *(1)*

**Supplements S2: Calculation of IMU-based gait features**

The IMU data were processed by, first, resampling the data to 100 samples per second and correcting the gyroscope offset. Next, a custom-made step-detection algorithm was applied to determine stance and swing phases in the 2MWT [1]. The following four gait features were calculated: speed [m/s], asymmetry, smoothness, and variability [s]. These four gait features were selected because they are reliable and represent different aspects of gait [1]. A description of the gait features is given below. The equations for the four variables are described in Table S1 in the appendix.

*Speed* is often used as a measure for the functional capacity and in stroke rehabilitation an indication of someone’s ability to walk in daily life, i.e., functional walking [2]. Computation of gait speed was done in four steps. First, a sensor-fusion algorithm was used to transform acceleration from a local to a global reference frame by combining the accelerometer and gyroscope data [3]. Second, the linear acceleration in the anterior-posterior direct was integrated once to determine the gait speed. Third, a Zero-Velocity Update was applied to set velocity to zero during stationary phases of walking, thereby reducing estimation errors [4]. Fourth, the corrected gait speed was integrated to determine position. This calculation enabled us to measure the total distance covered in the two-minute assessment, and thus, the gait speed.

*Asymmetry* is an expression of the asymmetry between the left and right side of an individual’s body during walking. The asymmetry ratio was calculated by dividing the stance-swing phase ratio of the paretic leg by the stance -swing phase ratio of the non-paretic leg. The stance -phase duration was calculated using the acceleration signal of the left and right foot sensor. In normal gait, there is typically a high degree of asymmetry, resulting in an asymmetry ratio close to 1. Evidence suggests that temporal asymmetry is negatively associated with preferred speed and motor recovery of the leg and foot [5].

*Variability* was expressed as the variability in stride time. The variability was calculated as the standard deviation of the time per stride. A lower gait variability score is related to a more stereotypical gait. An increase in variability appears to be an indicator for decline in locomotor function [6].

*Smoothness* characterizes the regularity of the movement patterns during walking. Smoothness was expressed as the index of harmonicity of the pelvis acceleration in the medio-lateral direction during walking. A higher index of harmonicity indicates a smoother gait. The smoothness appears to be associated with lower limb spasticity [7].

**Table S1. Equations of IMU-based gait features**

| Abbreviation | | Description | Formula |
| --- | --- | --- | --- |
|  | Speed | The gait speed during the 2MWT [m/s] | $\frac{Total distance}{120s} (2)$ |
|  | Asymmetry | The swing-stance phase ratio of the paretic foot divided by the swing-stance phase ratio of the non-paretic foot. | $\frac{swing-stance ratio Paretic}{swing stance ratio NonParetic} (3)$ |
|  | Variability | Standard deviation of the stride times of the left foot [s]. | $SD\left( time per stride \right)$ (4) |
|  | Smoothness | Index of harmonicity of the acceleration in medio-lateral direction. | Ratio of the amplitude of the dominant frequency to the sum of the first five super harmonics. |
| Abbreviations: 2MWT = Two minute walk test; AP: Anterior-Posterior; ML: Mediolateral; SD: Standard deviation; m = meters; s = seconds | | | |

**Supplements S3: Calculation of daily-life gait features**

After episodes of gait were identified in daily life, a custom-made step-detection algorithm was applied to count the total number of steps per epoch [1]. The total number of steps per day was calculated by summing all the number of steps per epoch and dividing this number by the wearing time. The wearing time was defined as the time the sensor was worn by the participant. Only data with a minimal wearing time of 8 hours was used. After the step detection, a sensor-fusion algorithm was used to transform acceleration from a local to a global reference frame by combining the accelerometer and gyroscope data per gait episode [3]. Second, the linear acceleration in the anterior-posterior direct was integrated once to determine the gait speed. Third, a Zero-Velocity Update was applied to set velocity to zero during stationary phases of walking, thereby reducing estimation errors [4]. Fourth, the corrected gait speed was integrated to determine position. This calculation enabled us to measure the total distance covered in the two-minute assessment, and thus, the gait speed. The average gait speed was calculated based on the overall speed throughout the assessment period, while the maximum gait speed was determined from the 95th percentile of the speed distribution. Measures of strides per day, average gait speed, and maximum gait speed were used to evaluate community walking.

**Supplements S4: Description of the Variational AutoEncoder**

Data processing for the Variational AutoEncoder (VAE) began by down sampling the data from an original frequency of 104 to 100 samples per second. The gyroscope data were then corrected using offset values from a static measurement. A step detection algorithm was applied to identify foot contacts and stance phases within the gait measurement [1]. To augment the amount of data available for training the VAE, all two-minute walk test data were segmented into epochs of 512 samples, starting in a stance phase with approximately 50% overlap. These epochs were first filtered using a first-order Butterworth bandpass filter with a 0.01–10 Hz range. The beginning of each epoch was normalized by setting the initial value to zero, and epochs were standardized; epochs with a z-score over five were deemed outliers and excluded. Finally, the data were normalized per dimension to a range between -1 and 1 using the minimal and maximal values measurable by the sensors (8g for the accelerometer and 500 rad/s for the gyroscope).

For model development, the VAE consisted of an encoder and a decoder, each with three mirrored convolutional layers. The model processed data in a 512 x 6 matrix format, representing triaxial acceleration and angular velocity, and compressed it into 12 latent features. The VAE aimed to minimize the Mean Squared Error and the Kullback-Leibler divergence to ensure the latent features were normally distributed and to enhance the model’s accuracy in representing the input data. More information is provided in the study of Felius et al (2024) [8]. The source code for the project can be accessed via: https://zenodo.org/doi/10.5281/zenodo.10878458.

**Supplements S5: Assumptions of the Ordinary Least Square Regression**

Evaluation of assumptions of 1) Linearity; 2) Normality of error terms; and 3) homoscedasticity of all significant predictors with Ordinary Least Squares.

**Fig S5.1 Assumptions of significant predictors at admission for strides per day six-months after stroke.**


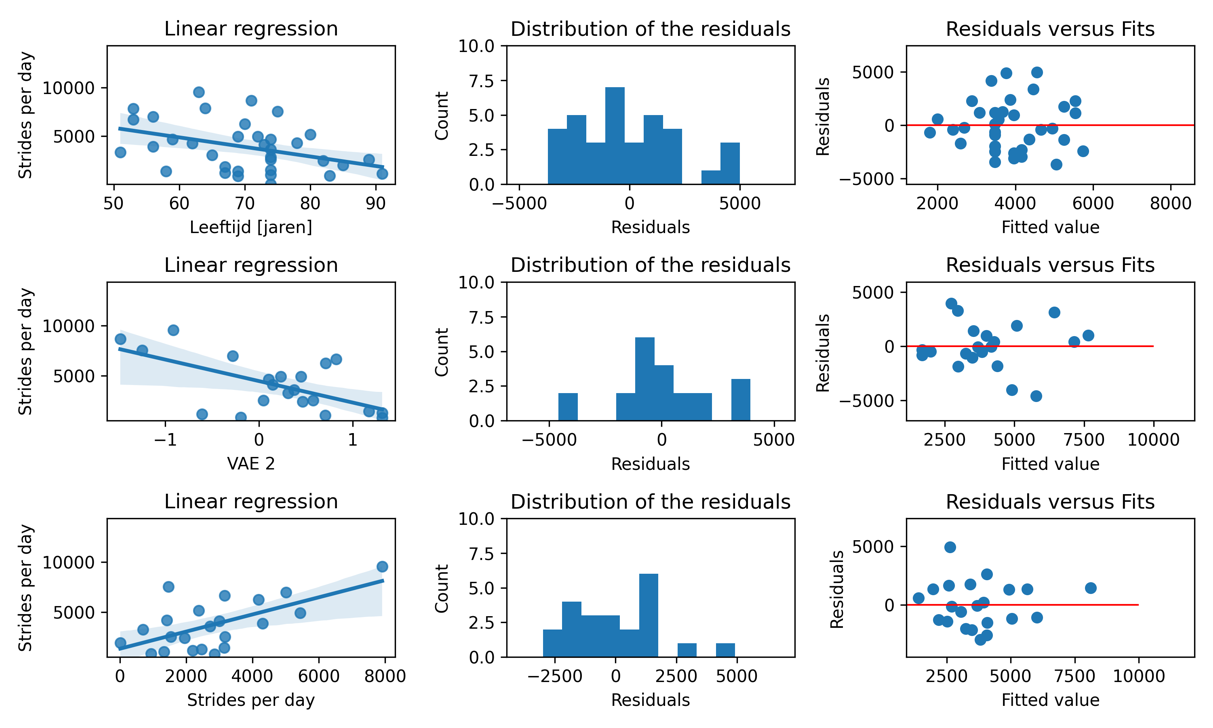


**Fig S5.2 Assumptions of significant predictors at discharge for strides per day six-months after stroke.**


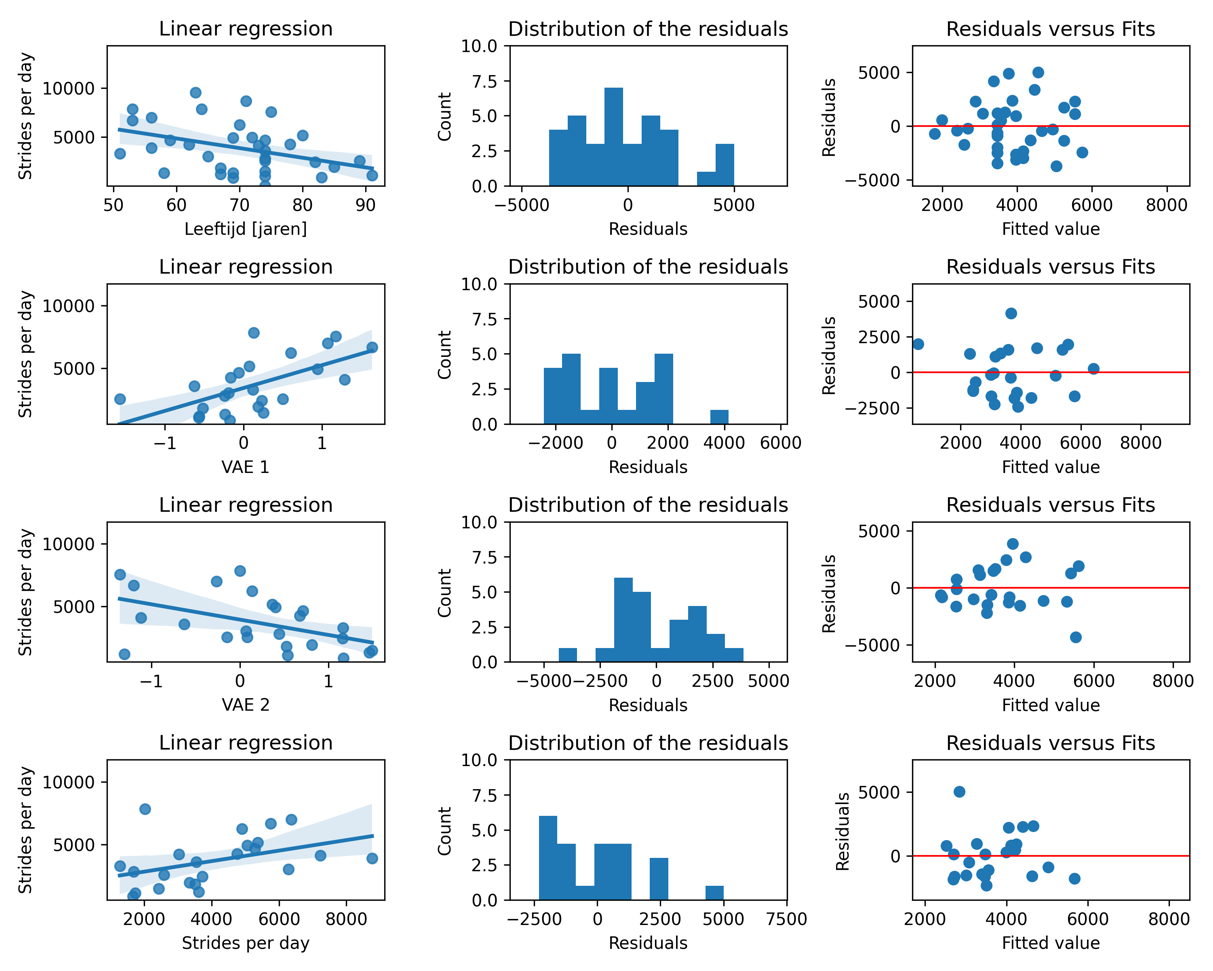


**Fig S5.3 Assumptions of significant predictors at admission for average gait speed at six-months after stroke.**


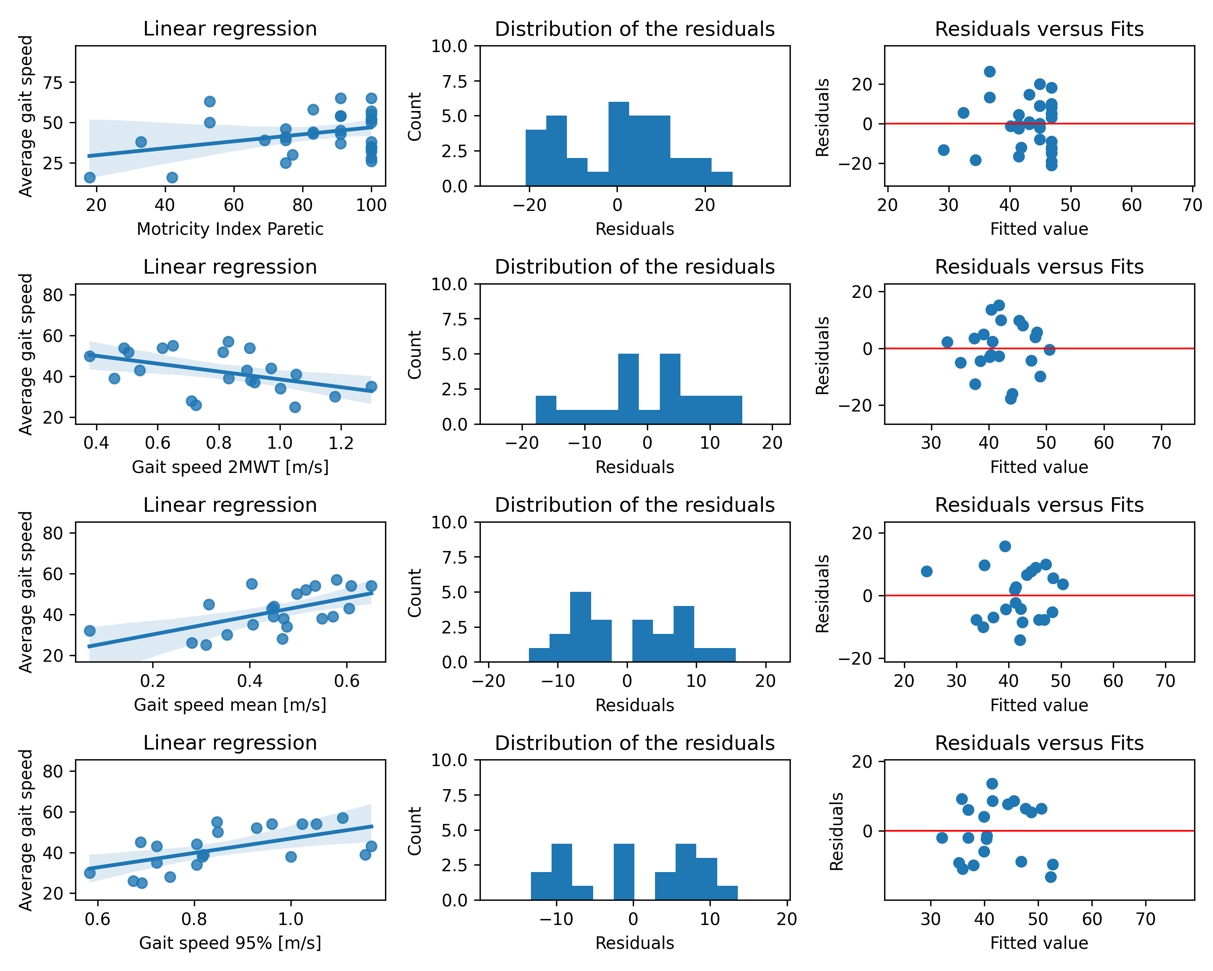


**Fig S5.4 Assumptions of significant predictors at discharge for average gait speed at six-months after stroke.**

**
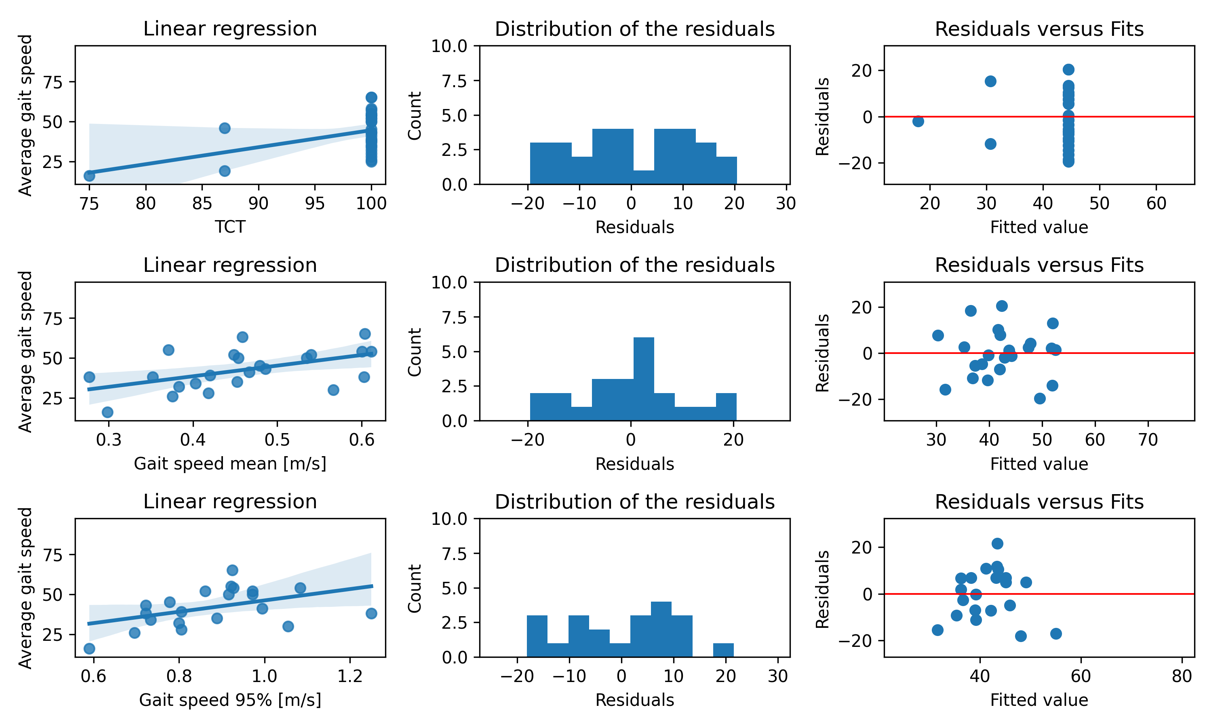
**

**Fig S5.5 Assumptions of significant predictors at discharge for the maximum gait speed at six-months after stroke.**


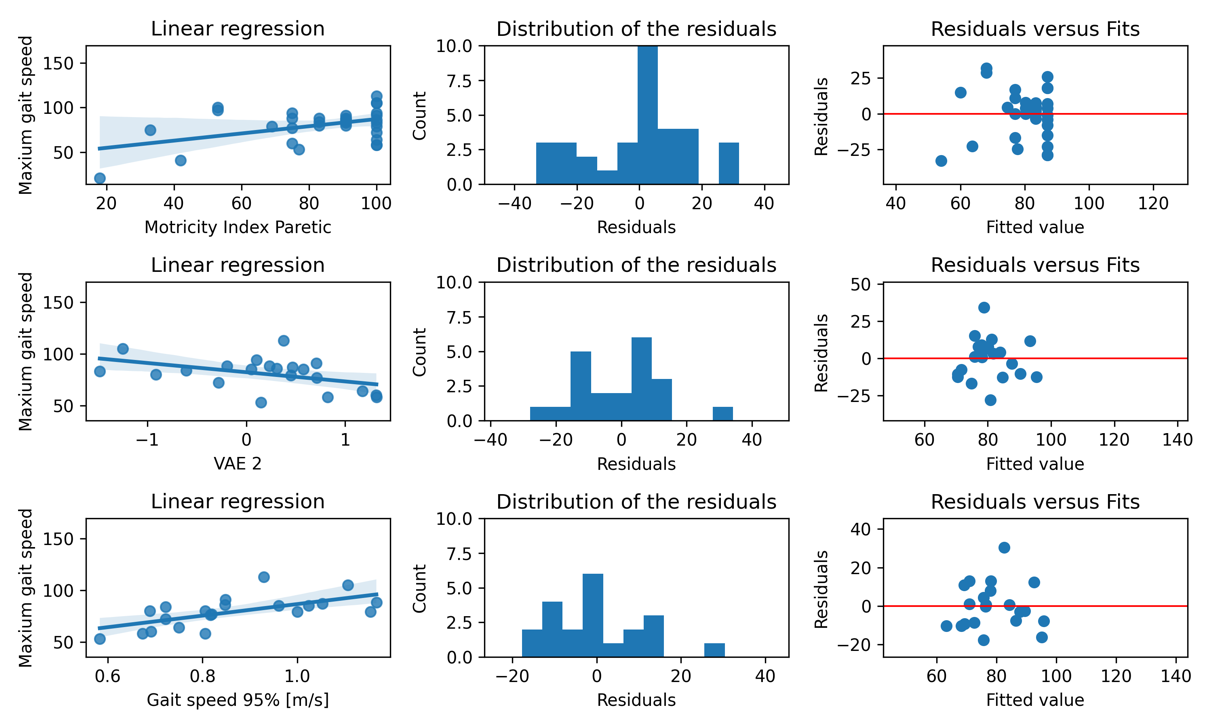


**Fig S5.6 Assumptions of significant predictors at discharge for maximum gait speed at six-months after stroke.**


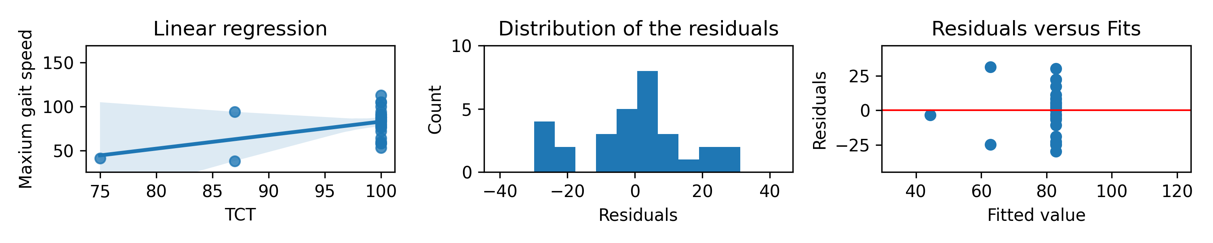


**Supplements S6: Correlation of the Multivariate Ordinary Least Square Regression**

**Table S6.1 Correlations of the dependent variables in the multivariate ordinary least square regression for the strides per day**

|  | Admission | | Discharge | | |
| --- | --- | --- | --- | --- | --- |
|  | Age [years] | VAE 1 | Age [years] | VAE 1 | VAE 2 |
| VAE 1 | -0.05 |  | -0.34 |  |  |
| VAE 2 |  |  | 0,27 | -0.30 |  |
| Strides per day | -0.36 | -0.14 | -0,27 | 0.54 | -4.81 |

**Table S6.2 Correlations of the variables in the multivariate ordinary least square regression estimating the average gait speed**

|  | Admission | Discharge |
| --- | --- | --- |
|  | Speed [m/s] | TCT |
| Average gait speed [m/s] | -0.42 | 0,46 |

**Table S6.3 Correlations of the variables in the multivariate ordinary least square regression estimating the maximum gait speed**

|  | Admission | |
| --- | --- | --- |
|  | MI Paretic leg | VAE 2 |
| VAE 2 | -0.0 |  |
| Maximum gait speed [m/s] | 0.02 | -0.26 |

**References**

1. Felius, R. A. W., Geerars, M., Bruijn, S. M., van Dieën, J. H., Wouda, N. C., & Punt, M. (2022). Reliability of IMU-Based Gait Assessment in Clinical Stroke Rehabilitation. Sensors, 22(3), 1-19. [908]. <https://doi.org/10.3390/s22030908>
2. Tasseel -Ponche, S., Delafontaine, A., Godefroy, O., Yelnik, A. P., Doutrellot, P. L., Duchossoy, C., Hyra, M., Sader, T., & Diouf, M. (2022). Walking speed at the acute and subacute stroke stage: A descriptive meta-analysis. Frontiers in neurology, 13, 989622. <https://doi.org/10.3389/fneur.2022.989622>
3. Madgwick, S. O. H., Harrison, A. J. L., Sharkey, P. M, Vaidyanathan, R. and Harwin, W. S (2013) Measuring motion with kinematically redundant accelerometer arrays: theory, simulation and implementation. Mechatronics, 23 (5). pp. 518- 529. ISSN 0957-4158 doi: https://doi.org/10.1016/j.mechatronics.2013.04.003 Available at https://centaur.reading.ac.uk/31540/
4. R. P. Suresh, V. Sridhar, J. Pramod and V. Talasila, "Zero Velocity Potential Update (ZUPT) as a Correction Technique," 2018 3rd International Conference On Internet of Things: Smart Innovation and Usages (IoT-SIU), Bhimtal, India, 2018, pp. 1-8, doi: 10.1109/IoT-SIU.2018.8519902.
5. Patterson, K. K., Parafianowicz, I., Danells, C. J., Closson, V., Verrier, M. C., Staines, W. R., Black, S. E., & McIlroy, W. E. (2008). Gait asymmetry in community-ambulating stroke survivors. Archives of physical medicine and rehabilitation, 89(2), 304–310. https://doi.org/10.1016/j.apmr.2007.08.142
6. Patel, P., Enzastiga, D., Casamento-Moran, A., Christou, E. A., & Lodha, N. (2022). Increased temporal stride variability contributes to impaired gait coordination after stroke. Scientific reports, 12(1), 12679. <https://doi.org/10.1038/s41598-022-17017-1>
7. Garcia, F. D. V., da Cunha, M. J., Schuch, C. P., Schifino, G. P., Balbinot, G., & Pagnussat, A. S. (2021). Movement smoothness in chronic post-stroke individuals walking in an outdoor environment-A cross-sectional study using IMU sensors. PloS one, 16(4), e0250100. <https://doi.org/10.1371/journal.pone.0250100>
8. R.A.W. Felius, M. Punt., N.C. Wouda, M. Geerars, R.B. Rutgers, S.M. Bruijn, S. David, and J.H. van Dieën (2024). Exploring Unsupervised Feature Extraction of IMU-based Gait Data in Stroke Rehabilitation using a Variational AutoEncoder. manuscript submitted for publication.
